# Compliance with Web Content Accessibility Guidelines in Ophthalmology Social Media Posts

**DOI:** 10.21203/rs.3.rs-3565120/v1

**Published:** 2023-11-08

**Authors:** Meghan Sharma, Serena Shah, Alexandra Gil, Laura Huertas, Elena Bitrian, Ta Chen Peter Chang

**Affiliations:** Bascom Palmer Eye Institute at the University of Miami Miller School of Medicine; Bascom Palmer Eye Institute at the University of Miami Miller School of Medicine; Florida International University; Bascom Palmer Eye Institute at the University of Miami Miller School of Medicine; Bascom Palmer Eye Institute at the University of Miami Miller School of Medicine; Bascom Palmer Eye Institute at the University of Miami Miller School of Medicine

**Keywords:** Social media, visual impairment, ophthalmology social media, web accessibility, Web Content Accessibility Guidelines, Americans with Disabilities Act

## Abstract

This is a cross-sectional analysis of publicly available Internet data to examine compliance to Web Content Accessibility Guidelines (WCAG) on patient education social media posts in ophthalmology. WCAG ensures web content accessibility for those with disabilities (including visual impairment). Social media posts were sampled from 10 ophthalmology patient education social media pages and 10 non-ophthalmology (cardiopulmonary) pages as the comparison group. Three independent reviewers graded the selected posts based on the WebAIM© WCAG 2 checklist adapted for social media posts. Validated accessibility standard labels: “0” for not meeting any standards, “1” for meeting bare minimum accessibility requirements, “2” for meeting legal accessibility requirements, or “3” for exceeding accessibility requirements. There were no significant differences between ophthalmology and non-ophthalmology posts in receiving high vs. low WCAG grades. 49% of ratings for ophthalmology social media posts showed no compliance with any WCAG. The most common reasons that ophthalmology posts failed to meet criteria were due to color and contrast issues (38.9%). Most ophthalmology social media posts had low WCAG scores, indicating poor compliance to WCAG. Because social media is highly visual, reduced compliance to WCAG may create barriers for low vision individuals to successfully access patient education social media content.

## INTRODUCTION

Social media is used by more than 4.2 billion people worldwide. [1] In the ophthalmology community, social media is utilized mostly for patient education purposes. [2] Prior studies have shown that Internet use is not impeded by visual impairment, which supports the notion that some or many consumers of ophthalmology-based patient education social media content may be visually impaired. [3, 4] Despite these findings, no study has analyzed compliance of social media posts intended for a visually impaired audience to web-based accessibility guidelines.

National guidelines have been published to increase accessibility for individuals with disabilities, including visual impairment. In 1990, the Americans with Disabilities Act (ADA) was passed to ensure “equal opportunity for persons with disabilities in employment, state, and local government services, public accommodations, commercial facilities, and transportation.” [5] Following the creation of the ADA, the Web Accessibility Initiative created the Web Content Accessibility Guidelines (WCAG) to enforce universal standards pertinent to web accessibility based on ADA objectives. [6, 7] WCAG 2.0, a more recent version of WCAG, is divided into 3 tiers of recommendations: level A reflects minimum accessibility, level AA signifies redress of the most common issues to meet legal requirements, and AAA refers to the elimination of all obstacles to exceed requirements. [8] These guidelines have been recommended by the United Nations to guarantee web-based accessibility for all. [9] In 2018, WCAG 2.1 was developed to address mobile accessibility and people with low vision, cognitive disabilities, and learning impairments. [5] Two years later, WebAIM©, a non-profit affiliated with Utah State University, created the WebAIM WCAG 2 checklist to condense the official WCAG 2.1 specifications. [7]

We hypothesize that, compared to non-ophthalmology posts, ophthalmology posts would have higher compliance with WCAG criteria. This study aims to investigate compliance to WCAG 2 among ophthalmology versus non-ophthalmology patient education social media posts using an adapted version of the WebAIM WCAG 2 checklist.

## METHODS

This study does not involve human subjects and involves freely available web contents in the public domain, hence an evaluation and approval by the Institutional Review Board were not required per institutional policy. Researchers performed a web-based analysis using Instagram because it is a more visual platform than other social media sites. [10] Online educational posts were sampled from ten ophthalmology and ten non-ophthalmology (cardiopulmonary) social media pages. Cardiopulmonary sites were chosen as the control group because researchers agreed that the intended audience would not be enriched with visually impaired individuals. [11] The first ten public, English-language pages that appeared after a search for ophthalmology awareness pages and the first ten public, English-language cardiopulmonary pages were chosen (Table 1). To find ophthalmology pages, the search terms “ophthalmology awareness,” “cataract awareness,” and “glaucoma awareness” were used. To find cardiopulmonary pages, the search terms “cardiopulmonary awareness,” “COPD awareness,” and “cholesterol awareness” were used. Five posts were systematically sampled for review by three independent reviewers during April of 2023. Independent reviewers graded all posts based on an adapted version of the WebAIM WCAG 2 checklist. Study designers obtained permission from WebAIM to adapt their original WCAG 2 checklist for the intention of analyzing social media posts. The resulting checklist is displayed in Table 2. This checklist contains only the criteria from the original checklist that are relevant to the analysis of social media posts.

Reviewers scored each post as “0” for not meeting any standards, “1” for bare minimum accessibility (A), “2” for accessibility meeting legal requirements (AA), or “3” for exceeding accessibility requirements (AAA). [7] Each reviewer began with a dichotomous evaluation of the post using A criteria, wherein failure to meet all criteria merited a zero and success in meeting all criteria merited continuance to the AA category. Then, failure to meet all AA criteria merited a 1 and success in meeting all criteria merited continuance to the AAA category. Finally, failure to meet all AAA criteria merited a 2, and success in meeting all criteria merited a 3. To determine the posts’ color contrasts (necessary to assess A, AA, and AAA criteria), each reviewer input the foreground and background colors into a WebAIM contrast checker. To determine the posts’ readability (necessary to assess the AAA criteria), each reviewer input the written content into a Flesch-Kincaid Grade Level calculator. The Flesch-Kincaid Reading Grade Level formula is commonly used by researchers to determine if health information is at a reading level that is appropriate for patients by measuring semantic and syntactic difficulty. [12] For each post, reviewers recorded which criteria of the adapted checklist were unmet, if any.

Descriptive statistics were applied to the variables of interest. An ordinal logistic regression was conducted to assess the relationship between increasing rating and category of post (ophthalmology or non-ophthalmology). To keep observations independent, the regression model used lowest of the three grades received by each post as its dependent variable in order to avoid overestimating any potential associations. Additionally, Fleiss’s Kappa was calculated to determine the inter-rater reliability among all three independent graders.

## RESULTS

**Figure 1:**
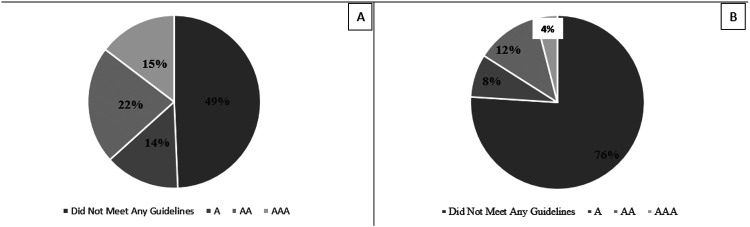
Overall ratings and lowest ratings for ophthalmology social media posts Accessibility ratings of ophthalmology social media posts according to guidelines of Web Content Accessibility Guidelines (WCAG 2) adapted checklist. A= Bare Minimum Accessibility; AA = Accessibility Meeting Legal Requirements; AAA = Exceeds Accessibility Requirements. Figure 1A. Distribution of all ratings for 50 ophthalmology posts obtained from 3 independent graders (N=150) Figure 1B. Distribution of lowest ratings for 50 ophthalmology posts. The lowest rating of the three ratings obtained from three graders for each of the 50 ophthalmology posts was used (N=50).

**Figure 2:**
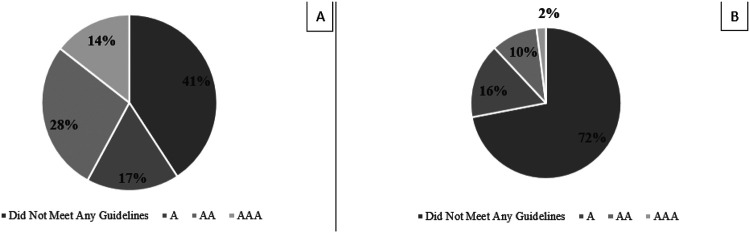
Overall ratings and lowest ratings for non-ophthalmology social media posts Accessibility ratings of ophthalmology social media posts according to guidelines of Web Content Accessibility Guidelines (WCAG 2) adapted checklist. A= Bare Minimum Accessibility; AA = Accessibility Meeting Legal Requirements; AAA = Exceeds Accessibility Requirements. Figure 2A. Distribution of all ratings for 50 non-ophthalmology posts obtained from 3 independent graders (N=150) Figure 2B. Distribution of lowest ratings for 50 non-ophthalmology posts. The lowest rating of the three ratings obtained from three graders for each of the 50 non-ophthalmology posts was used (N=50).

**Figure 3:**
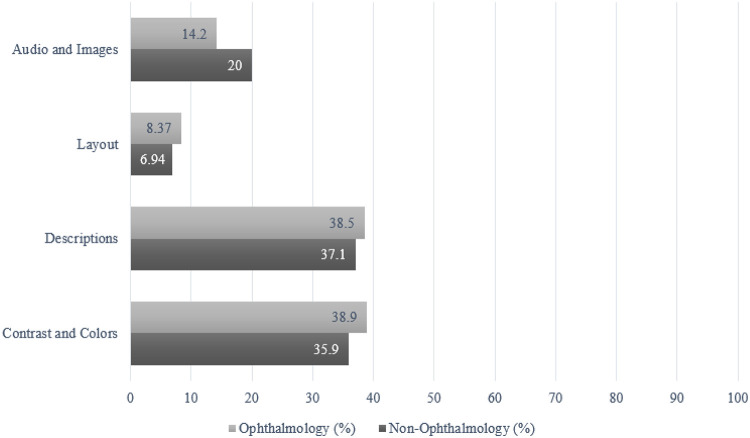
Failing criteria for ophthalmology and non-ophthalmology social media posts determined by three independent reviewers (N=300) Independent reviewers recorded the criteria from the Web Content Accessibility Guidelines (WCAG) adapted checklist that caused a post to fail. The failing criteria were divided into four categories: audio and images, layout, descriptions, and contrast and colors. Reviewers could choose more than one failing criterion for each post.

A total of 100 posts (50 ophthalmology and 50 non-ophthalmology) were reviewed by the 3 reviewers, resulting in a total of 300 ratings. Among them, the most common rating received by both ophthalmology posts (49%, Figure 1) and non-ophthalmology posts (41%, Figure 2) was 0 for not meeting any WCAG standards. All reported reasons for why a post did not pass criteria are displayed in Figure 3 and are expressed as four criteria categories: contrast and colors as criteria 3, 5, 6, and 9 from the checklist; descriptions as criteria 1, 12, 13, and 14; layout as criteria 2, 8, 10, and 11; and audio and images as criteria 4, 7, 15, 16, and 17. The most common category that ophthalmology posts failed was contrast and colors (38.9%) followed by descriptions (38.5%), audio and images (14.2%), and layout (8.37%). The most common category that non-ophthalmology posts failed was descriptions (37.1%), followed by contrast and colors (35.9%), audio and images (20.0%), and layout (6.94%).

**Figure 4:**
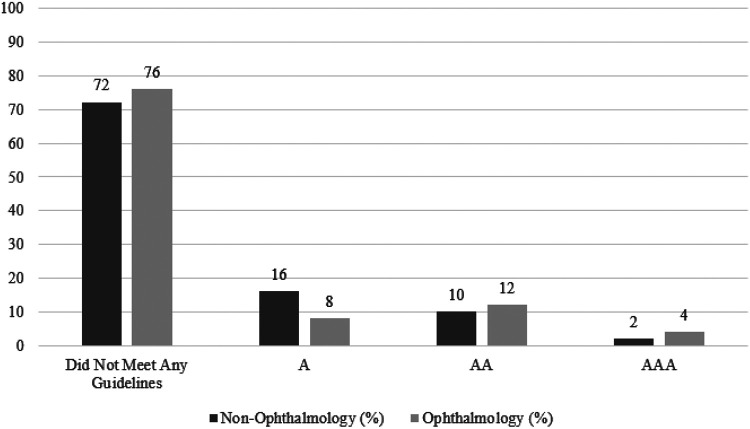
Lowest WCAG ratings for non-ophthalmology and ophthalmology social media posts (N=100) Distribution of the lowest ratings obtained from the three graders for ophthalmology and non-ophthalmology posts. An ordinal logistic regression model comparing the lowest overall ratings for ophthalmology posts to the lowest overall ratings for non-ophthalmology posts showed no significance (p=0.80).

The lowest rating for ophthalmology posts was “0” for not meeting any guidelines for 76% of posts, A for 8% of posts, AA for 12% of posts, and AAA for 4% of posts. These results were compared to the lowest ratings for non-ophthalmology posts, which were “0” for not meeting any guidelines for 72% of posts, A for 16% of posts, AA for 10% of posts, and AAA for 2% of posts (Figure 4). The model showed no significance, suggesting that the ophthalmology social media posts were not more likely to receive higher ratings on accessibility than non-ophthalmology posts (p=0.80), in contrary to our hypothesis. However, there was only fair agreement among the ratings given by the reviewers (*κ* = 0.21, p<0.0001), suggesting low inter-rater reliability.

## DISCUSSION

Social media has played a growing role in patient education efforts. [13] Although many low vision individuals use social media, they likely encounter accessibility challenges. To the authors’ knowledge, this study is the first to analyze compliance to WCAG for ophthalmology social media posts and found no evidence that posts that were intended for a visually impaired audience met guidelines compared to those that were not. Our findings showed that 49% of overall ratings for ophthalmology social media posts did not meet any WCAG standards, which is consistent with other studies examining general WCAG compliance among web pages. A 2023 study by Utah State University tested the top one million web homepages and found that 96.3% were not compliant. [14] Another study found that the majority of e-government websites in India did not meet Level A standard with WCAG 2.1. [15] Our findings suggest that more ophthalmology posts than non-ophthalmology posts did not adhere to any WCAG guidelines, with contrast issues being the most common reason for failure. Similarly, a study examining web accessibility of the library webpages of top-ranking United States post-secondary institutions found that approximately half of errors were categorized as contrast errors, directly impacted those with visual impairments. [5] Based on these findings, enhancing contrast either at the content creator or consumer’s level may significantly improve the accessibility and educational impact of these ophthalmology posts to the intended audience.

This overall poor WCAG compliance among ophthalmology posts may be explained by several factors. First, content creators may not be aware of WCAG standards. Despite the growing availability of accessibility information over the past two decades, website accessibility has not improved appreciably. An analysis by Swallow et al. contributed some of these findings to external factors, such as client and organizational attitudes to web accessibility or difficulty in enforcing accessibility legislation. [16] Moreover, creators may experience difficulties in utilizing the accessibility information provided by tools, guidelines, and resources. One study analyzing 17 students taking part in a web accessibility course concluded that WCAG is “far from testable for beginners” due to difficulty in comprehending the language used in guidelines. [17]

Tools such as the WebAIM WCAG 2 checklist were created to simplify the official WCAG 2.1 specifications into a more usable checklist for web creators; however, this study highlighted several limitations of the checklist. Many of the WebAIM criteria are subjective, such as “descriptive and informative post title,” which reviewers may grade differently. In our study, designers attempted to decrease subjectivity and bias by having multiple reviewers grade posts; however, there was low inter-rater reliability, suggesting that grading of posts still varied despite using a controlled, stepwise evaluation algorithm. Additionally, the WebAIM WCAG 2 checklist may not be the most suited for content addressing a visually impaired audience. For instance, there are no criteria on the checklist referring to type of font used, as some fonts may appear more obscured or difficult to read than others. This may impact accessibility of the post for a visually impaired individual. Finally, the checklist was not the most suitable for analyzing social media posts, as many criteria from the original checklist needed to be omitted because they were not relevant for social media posts. For example, several original criteria specify keyboard function requirements, but keyboard functionality is a feature controlled by Instagram rather than post-creators. [7] Study creators concluded that this criterion and other criteria not applicable to singular social media posts should be omitted in the adapted WebAIM WCAG checklist that was used for this study.

This study has several limitations. We had assumed that the content creators aimed to educate the public, while the main incentive for for-profit content creation is typically to exert and widen one’s digital influence. Hence, there may not be sufficient incentives for WCAG compliance as this may or may not increase the number of “views,” “shares,” or “likes” that signifies a post’s popularity. Also, this cross-sectional study does not speak toward the overall trend of WCAG compliance, nor can we generalize on the variability of WCAG compliance using our modestly-sized sample against the vast web content that currently exists on the Internet. Future studies could amend study design to see if WCAG compliance influences a post’s popularity, while a subsequent study may create and validate a new tool for analysis of social media posts to WCAG guidelines, since the original WebAIM WCAG checklist is tailored specifically to online websites. Finally, increasing the number of posts examined in a future study may provide greater insight to determine generalizability.

## CONCLUSION

Social media is an important tool in the dissemination of information for patients in ophthalmology. This study is the first to analyze compliance to WCAG for ophthalmology social media posts and found that there was overall poor compliance, potentially limiting the content accessibility for visually impaired consumers. Raising awareness in WCAG compliance can potentially improve ophthalmology patient educational access via social media for the intended audience.

## Data Availability

All data used in this study is publicly available.
